# Assessing Dementia in Resource-Poor Regions

**DOI:** 10.1007/s11910-012-0300-9

**Published:** 2012-08-05

**Authors:** Gladys E. Maestre

**Affiliations:** 1 Laboratory of Neurosciences, University of Zulia, Edificio del Instituto de Enfermedades Cardiovasculares de la Universidad del Zulia, Primer Piso, Av Universidad diagonal al MACZUL, Maracaibo, 4002 Zulia Venezuela; 2 Departments of Psychiatry and Neurology, and Gertrude H. Sergievsky Center, Columbia University, PH 19 Room 315, 622 West 168th Street, New York, NY 10032 USA

**Keywords:** Developing countries, Low-to-middle-income countries, Resource-poor, Population demography, Elderly, Dementia, Prevalence, Diagnosis, Capacity building, Challenges, Obstacles, International partnerships, Cost–benefit analysis, Economics, Ethics

## Abstract

The numbers and proportions of elderly are increasing rapidly in developing countries, where prevalence of dementia is often high. Providing cost-effective services for dementia sufferers and their caregivers in these resource-poor regions poses numerous challenges; developing resources for diagnosis must be the first step. Capacity building for diagnosis involves training and education of healthcare providers, as well as the general public, development of infrastructure, and resolution of economic and ethical issues. Recent progress in some low-to-middle-income countries (LMICs) provides evidence that partnerships between wealthy and resource-poor countries, and between developing countries, can improve diagnostic capabilities. Without the involvement of the mental health community of developed countries in such capacity-building programs, dementia in the developing world is a disaster waiting to happen.

## Introduction

Dementia disrupts normal functioning of affected individuals and their families, imposing significant social and economic burdens. The problem is especially severe in low-to-middle-income countries (LMICs), where dementia is the most important independent contributor to disability in the elderly [1] and resources to diagnose and treat dementia are limited. By the mid-21st century, 78 % of the world’s elderly population will reside in LMICs, with expected concomitant increases in dementia cases [2]. Prevalence of dementia is relatively low in some LMICs, but other regions have a prevalence similar to or higher than that in developed countries [3]. For example, Latin America and the Caribbean (LAC) have the world's highest prevalence of dementia (8.5 % in subjects ≥60 years vs. 6.4 % in the U.S.) [3–5].

It is in resource-poor regions with a high prevalence of dementia that cost-effective health care targeted at vulnerable populations is most needed. However, dementia care is problematic, even in high-income countries, many patients with dementia remain undiagnosed and, therefore, untreated [6, 7•]. The governments of LMICs and international healthcare agencies have tended to disregard chronic conditions of the elderly, due to more immediate and widespread problems of malnutrition and communicable diseases. However, the World Health Organization (WHO) recently released guidelines for increasing treatment of dementia in LMICs [8] and urged governments to consider dementia as a public health priority [9••]. Developing effective approaches for diagnosis is the first step toward providing adequate services for dementia sufferers and their caregivers. In this article, I review strategies used to diagnose dementia in resource-poor regions, discuss the particular challenges associated with those assessments, describe some recent strategies for developing and improving diagnosis in poor countries, and consider how diagnosis of dementia might be improved in the future.

## Prevalence of Dementia in the Developing World

There are many estimates of age-specific or age-and-gender-specific dementia prevalence in the developed world, based on epidemiological studies. However, WHO identified only 64 such studies in 14 developing countries [9••]. Where such data are not available, estimates have been derived from a Delphi consensus of experts [10]. Furthermore, the age-specific and age-and-gender-specific estimates for developing countries are often compared with estimates for developed countries, despite different population structures. Therefore, estimates of the prevalence of dementia in the developing world should be viewed with caution.

Most estimates of the prevalence of dementia in people older than 60 years fall between 5 % and 7 % [3]. Variation in actual prevalence—as opposed to variation in detected dementia—is influenced by a number of factors. For example, low prevalence in sub-Saharan Africa (2.1 %) might be due to selective mortality of people younger than 60 years [11]. High prevalence in LAC (8.5 %) might be related to the high occurrence or confluence of genetic and/or environmental risk factors [5]. For example, increased mobility of individuals and families has spread genetic isolates with an extremely high prevalence of dementia, such as those with early-onset Alzheimer´s disease in Medellin, Colombia [12] and Huntington’s disease in Maracaibo, Venezuela [13]. The fact that expression of the disease occurs after reproductive age might also contribute to its spread. Finally, in consanguineous marriages, which are relatively common in some resource-poor areas, dementia in either or both parents increases the risk of having children who will be affected in the future [14].

Population demography and other factors play an important role in determining the prevalence of dementia. Both prevalence and incidence of dementia increase exponentially with age, making age the main risk factor for the disease [9••, 15]. The number of people at risk of dementia in the developing world will increase rapidly during the next 50 years, because the population and proportion will grow faster for the elderly (≥60 years) than for other age groups [16]. In India, for example, the population of individuals ≥80 years will increase from 5.4 million in 2001 to 32 million in 2051 [17]. In addition to aging populations, gradual improvements in health care in developing countries are probably increasing the time that affected individuals survive with dementia [18]. Other factors that might affect the incidence of certain types of dementia—for example, cardiovascular risk factors, such as obesity, metabolic syndrome, and sedentary life—are also escalating much more rapidly in developing than in developed countries [19–22]. In consequence, the dementia burden in the developing world is expected to increase significantly.

## Diagnosing Dementia

Diagnosis of dementia and accurate determination of subtype early in the course of the disease are crucial for optimal clinical care and management. Traditionally, dementia has been defined as a global impairment of intellectual function that requires anatomopathological examination to provide accurate diagnosis and differentiation among subtypes [23–25]. However, research involving biomarkers and neurogenetics now allows identification of patients years, perhaps decades, before the diagnosis of dementia is made, such as in presymptomatic Alzheimer´s disease [26].

The National Institute on Aging and the Alzheimer´s Association recently released revised criteria for all-cause dementia [27], which include neuropsychiatric or cognitive symptoms, rather than just cognitive deficits, that interfere with daily functioning, represent a decline from previous levels, and are not explained by delirium or major psychiatric disorders. A new category was added, primarily intended for research purposes: dementia due to possible or probable Alzheimer´s disease with evidence of the Alzheimer pathology. The new category requires that a subject meeting the clinical criteria for Alzheimer´s disease also exhibits changes in biomarkers related to the current understanding of the pathophysiology [28]. The revised criteria for Alzheimer´s disease include a predementia stage, in which a subject is asymptomatic but shows evidence of the underlying brain pathology specific for Alzheimer´s disease. These new criteria will be of limited use in the developing world, because the ability to detect relevant biomarkers and pathologies is low, due to the high cost of testing and the lack of infrastructure.

To establish a diagnosis of dementia, an evaluation focuses on, as a clinical syndrome, cognitive performance and behavior of the subject, changes in the activities of daily living, and exclusion of other possible causes. Diagnosing dementia, particularly in the early stages, is neither simple nor straightforward. The clinical interview is considered to be an art. The first step is usually to elicit a complete history of signs and symptoms, recalled and recounted by the patient and/or an informant. A highly skilled interviewer structures the interaction as a conversation, establishing rapport and confidence and enabling the patient to fully disclose even embarrassing details. Neuropsychological, neuroimaging, and other diagnostic tests are used to distinguish pathological changes within the brain that characterize different dementia syndromes. Interpretation of the clinical interview and test results requires training and often varies among individuals making the judgment.

The clinician uses as many tools as possible—clinical examination, laboratory tests, neuroimaging, genetic testing—to determine the underlying pathology or type of dementia and establish the best therapeutic course as early as possible. However, the diagnostic profiles of different dementia subtypes are not 100 % distinct, and the correlation between clinical and pathological diagnoses is not perfect, even when based on the most advanced tools [18]. Furthermore, diagnostic profiles exhibit a great deal of heterogeneity, even among patients who share the same pathology, and some are frankly atypical [29]. Because of these challenges, accurate diagnosis requires an experienced clinician, supported by an array of complex tools [7•, 30]

## Obstacles to Diagnosis of Dementia in Poor Populations

Many factors contribute to limited diagnosis of dementia in resource-poor areas (Table 1). Three major obstacles are (1) low health literacy, (2) limited access to health care, and (3) the stigma associated with dementia. The extent of health illiteracy, including ignorance of the connection between behavioral, cognitive, or physical signs and the disease [31], is difficult to estimate but very important, as is lack of awareness that appropriate care/treatment is available [32]. Surprisingly, such ignorance often exists in educated individuals and even in healthcare professionals [33]. Lack of knowledge in healthcare workers particularly affects their ability to recognize early signs of dementia. Many people in developing countries believe that the characteristic signs of dementia are part of normal aging or a nonpathological deviation [34]. Improving health literacy is the goal of health promotion and education programs, which are often given low priority in resource-poor settings. On the other hand, the impact of a lack of specific or scientific knowledge should not be overestimated. Cultures in developing countries often have knowledge about signs, based on common sense and traditional practices, that informs choices about care/treatment [35].

**Table 1:** Likely causes of limited dementia diagnoses in resource-poor areas

- Initial symptoms are subtle and fluctuating
- Inability to recognize cognitive, behavioral, or functional impairment as a consequence of dementing illness
- Accommodation, denial, and rationalization of symptoms as due to aging and adverse circumstances
- High tolerance for health problems in elderly individuals
- Person with early symptoms does not feel the need to seek treatment
- Relatives do not believe symptoms are treatable
- Demoralization regarding medical assistance, due to ignorance of available treatments
- Do not know where to go for diagnosis
- Family is afraid of psychiatric stigma (demented is often equated to being crazy)
- Physician underemphasizes the importance of symptoms
- Perception that physicians will miss the diagnosis and will make the family waste time and money in apparently unnecessary tests
- Costs of diagnosis in terms of effort, time, economic costs

Limited access to health care is a universal problem in diagnosing dementia in LMICs. Geriatric care is generally minimal, and there is virtually no concept of continuing care for aging adults [36–38]. In addition to inadequate numbers of personnel and facilities, the costs involved in accessing services and long waits for appointments are common barriers. If an elderly person has to travel a long distance to receive a diagnosis and treatment, he or she must weigh the likelihood of improvement against the difficulties and costs of travel [39]. Travel where public transportation is not adequate for people with physical disabilities can be a limitation. Additionally, the patient and his or her caretakers might fear that there is no effective treatment, that treatment will require expensive drugs, or that the patient might have to stay at an asylum or hospice. Any or all of these factors might prevent a potential patient or caregiver from seeking medical attention, particularly in the case of a chronic condition that has established slowly, such as dementia.

The social stigma associated with mental health problems is a barrier to diagnosing dementia [40–42]. When dementia is medicalized and acquires the label of a disease, the social value of the individual and his or her family often suffers [11, 35]. Even when it is not labeled as a disease, signs of dementia are a fairly common condition in many resource-poor regions, and most people have a negative expectation of recovery [11]. Behavioral changes associated with dementia sometimes elicit abuse, neglect, or loss of family members’ respect [40]. Even healthcare workers are sometimes guilty of a negative attitude toward dementia patients [41–43].

In addition to health illiteracy, poor access to healthcare, and social stigma, diagnosing dementia in poor populations remains problematic for a number of reasons. Although the clinical features and complications of dementia have been relatively well defined [44], expression of behavioral and cognitive signs and symptoms might be modulated by socioeconomic conditions [41], possibly accounting for some of the phenotypic heterogeneity of various subtypes. Long-term studies are needed of the effects of lifestyles in developing countries on each of the dementia subtypes [45]. Although some genetic and environmental modifiers have been identified [46–48], it is still not possible to predict the likely course of the illness, even when dominant mutations are present [45]. The effects of concomitant comorbidities, such as hypertension and diabetes, are not clearly understood, particularly when untreated or uncontrolled for most of the life of the patient [49].

Practical approaches to overcoming some obstacles to diagnosis of dementia have been addressed in the context of minority populations [50•] (Table 2). There are other obstacles, particularly to early diagnosis of dementia, that are beyond the scope of this article [51]. However, it is important to recognize that economic crises, epidemics, famine, war, displacement, and natural disasters have devastating effects on populations in resource-poor countries, which relegate the health of elderly individuals to low priority [52, 53]. Barriers to improvement of dementia diagnosis and care are similar to those confronting mental health services [54]. Overcoming those barriers will require changes in politics, leadership, planning, advocacy, and participation [55].

**Table 2:** Options for and challenges to confronting critical issues in the diagnosis of dementia among minorities that are applicable to resource-poor regions

Issues	Options	Challenges
Need culture-unbiased instruments	Adapt existing tests	Comparability
	Develop new instruments	Alteration of psychometric properties
		Need local normative values
Undetermined value of informant data	Identify key informant	Stigma
	Home-based interview	Oversimplification of complex social relationships and environments
Assessment of daily functioning	Skilled clinician	Time consuming
	Home-based interview	Specific to setting
Interpretation of conflicting information	Multidisciplinary consensus on diagnosis	Experienced clinical judgment
	Algorithm diagnosis	Intimate knowledge of culture

Inarguably, it is crucial to reliably diagnose individuals with dementia in order to carry out clinical trials for treatments, to advance genetic studies, and to proceed with epidemiological studies that allow the establishment of specific risk factors. However, because there is no definite marker for the disease (except when relatively rare dominant genetic alterations are present and consistent with symptomatology), population screening and preclinical diagnosis in general populations are not yet cost effective and have not been implemented even in wealthy countries. Even screening for the ApoE-4 allele, the strongest risk factor for Alzheimer´s disease in most populations, is not generally recommended because of prognostic uncertainty [56]. In contrast, screening populations at high risk for dementia (e.g., elderly individuals with a medical history of risk factors for dementia) might be justifiable, but systematic recommendations have not been developed [57]. Nevertheless, the benefits of early diagnosis, coupled with proper care and caregiver support, outweigh the costs of diagnosis in developing countries [7•]. Although the only specific treatment for dementia, cholinesterase inhibitors and/or memantine, is available to a relatively small number of patients, primarily in wealthy countries [58], therapeutic interventions are currently being tested. Now is the time to prepare for their availability.

## Human Resource Capacity for Diagnosing Dementia in LMICs

While the number of specialty centers for dementia care has grown considerably in wealthier countries, apart from a few evolving areas [59–61], facilities for diagnosis and treatment of dementia are scarce in LMICs [62].

There is also a paucity of specialists devoted to health care of the elderly and to brain and mental health disorders [63–68]. Neuropsychologists and biometricians who are competent to administer standardized tests to local populations and interpret the results are rare, so normative values that can be appropriately applied by clinicians in diagnosing dementia are even rarer [51, 69]. The Kyoto Declaration of Alzheimer´s Disease International (ADI), on the basis of the recommendations of WHO 2001 [70], recommended integrating dementia outreach into existing primary health care, and WHO developed evidence-based guidelines for management of dementia by nonspecialists in LMICs [8]. Nevertheless, most primary care physicians and even many specialists in developing countries do not receive suitable training to diagnose dementia and its subtypes [40], despite efforts to remedy this situation during the past few years [71].

Building capacity to recognize and accurately diagnose dementia in LMICs, by training local healthcare personnel, is necessary to expand access to and improve mental health care [7•, 64]. To date, most initiatives for training healthcare workers in dementia diagnosis have depended largely on face-to-face interactions [72, 73], but the role of distance and Web- or mobile-telephone-based training is increasing [74–76]. However, none of the Web-based programs appear to offer training in different languages or to include specific information on assessment of individuals with low levels of education. Capacity-building efforts should also aim to improve referral pathways and promote multidisciplinary care of dementia patients, which will require training of nurses, social workers, and other healthcare personnel, in addition to specialists, such as geriatricians, neurologists, and psychiatrists.

Continued medical education in much of the developing world is currently subsidized by pharmaceutical industries [77], without strict regulations on content or selection of presenters. It is not surprising that many educational and dissemination programs on dementia are biased toward pharmacological approaches and downplay nonpharmacological strategies for managing behavioral symptoms [78]. Improvement and expansion of those programs to include dementia require input by both large international agencies and academia. Little is likely to be accomplished without their recognition of dementia as a public health problem in poor populations, with emphasis on prevention of risk factors, early diagnosis, and improved caregiving.

## Economic Issues Affecting Diagnosis of Dementia in LMIC

Economic issues are critical to the control and management of dementia in LMICs [79]. Economic issues also drive the perceived need for early diagnosis of dementia and differentiation of subtypes [7•]. To develop appropriate and effective policies, resource-poor countries require information about the overall prevalence and clinical burden of dementia in their populations, as well as cost–benefit analysis of accurate diagnosis and improved health care.

The human capital contributed by the most able elders, particularly in early stages of the aging process (55–70 years), is critically needed in developing countries; their experience contributes significantly to both economic productivity and family stability [80]. One way to visualize the economic costs of dementia is to consider the potential years of healthy life lost to disability (loss of function) and to premature death, through the concept of disability-adjusted life years (DALYs) [81]. Economic impact of disease also can be assessed by quality-adjusted life year (QALY), which combines duration and quality of life [82]. Since most of the dementia cases diagnosed in the developing world are of moderate to advanced severity, the price paid by society, either in DALYs or QALYs, is very high.

Cost-comparison analyses use the costs of diagnosis and of pharmacological and psychosocial interventions and other relevant costs to estimate the economic benefits of early diagnosis and early interventions [58, 83, 84]. A lack of mortality and natural history data make such calculations difficult for LMICs. Although not entirely projectable to resource-poor areas, a few general conclusions have been drawn from studies of developed countries [83–85]. First, the net benefits of early identification and intervention exceed costs, with significant savings expected. Second, the earlier the diagnosis and intervention, the greater the net benefits. Finally, caregiver interventions at earlier stages have beneficial economic effects. However, these analyses were not based on direct observations or randomized controlled trials, and no such analyses have been made for countries in the developing world.

## Ethical Issues Related to Diagnosis of Dementia in LMIC

Perceptions of the nature of disease, what social scientists call *social representations* of disease [86], vary widely among different populations and ethnic groups, resulting in the need to adapt educational and counseling programs related to dementia [42]. Low levels of education and health literacy, poor access to health care, and the fact that many elderly subjects live within extended families pose ethical dilemmas that need to be discussed with healthcare workers before an early detection program is implemented in a resource-poor community.

The key ethical principles guiding the process of disclosure of diagnosis at early stages in the disease are beneficence, respect for the autonomy of patients, and justice for patients [87, 88]. In other words, the clinician weighs the possible benefits and harm of disclosure, and this process is related to perceptions about the ability of the patient to understand and/or retain information, the potential psychological impact, and beliefs about the efficacy of treatments [89]. Respecting the patient’s autonomy is based on the notion that every individual has a right to control his/her life, as stated in Black´s Alzheimer Bill of Rights [90]. The principle of justice involves hearing the voice of patients, acknowledging their vulnerabilities, giving them a chance to decide important issues while decision making is still possible, and not marginalizing them.

Unfortunately, no one has studied preferences or the impact of diagnostic disclosure in developing countries among patients, caregivers, and health professionals. It could be difficult to persuade family members that some signs and symptoms in their elderly relatives are due to a disease of the brain, and not just to the process of aging. The belief that an affected individual will become seriously dependent can have disastrous effects, including marital breakdown, suicide by the patient, and social stigma for family members [11, 91]. Attitudes toward dementia diagnoses are often based on religious or spiritual beliefs [92]. These issues are even more sensitive when a dominant gene is implicated in the dementia, possibly resulting in discrimination and/or exclusion [93]. Until research on the impact of disclosure is conducted in developing countries, the decision to disclose a diagnosis must depend on the probable impact on a particular patient: “If a patient is likely to benefit, he or she should be told, but if benefit is not likely, or if disclosure is instead apt to bring about an adverse reaction, the disclosure is not advised” [94].

## The Role of International Partnerships in Improving Diagnosis of Dementia in Resource-Poor Countries

Partnerships between wealthy countries and LMICs (North/South partnerships) and partnerships between LMICs that have expertise in dementia and adjacent countries with no such expertise (South/South partnerships) are two strategies for improving diagnosis of dementia in resource-poor populations. However, those strategies have been implemented parsimoniously, and with few exceptions, large international funding organizations have shown little interest in partnering with LMICs to build capacity for dementia diagnosis.

Some genuine partnerships encompassing research and capacity building for delivery of services in developing countries have been extremely successful, not only in terms of scientific productivity, but also in the creation of services and dementia care options [95–97]. The research components of these partnerships have primarily been funded through governments and charitable organizations of high-income countries (North/South partnerships). Several agencies, including the Fogarty International Center of the NIH, have developed a program called “Brain Disorders in the Developing World: Research Across the Lifespan.” The program includes research and capacity building and has funded several initiatives devoted to dementia.

Only 8 % of mental health projects to date involved collaboration between LMICs, as compared with 30 % that involved collaboration with high-income countries [98]. Capacity building has less often been included in South/South partnerships, which have generally been supported by a variety of sources, including individual donors and charitable organizations. In most cases, the resulting programs have been expected to be self-sustaining after the development of services. However, the potential of South/South collaborations is beginning to emerge as a significant mechanism for empowering countries in resource-poor areas. Such partnerships can provide alternative sources of financial and technical assistance [99], like the collaboration in which Indian universities have provided virtual classes for medical staff and online consultations in Africa [99]. However, funding allocated for research on mental and neurological conditions in LMICs is vastly insufficient, and lobbying of international organizations by coalitions of LMICs might be needed to gain external support for South/South partnerships.

The main goals of international partnerships should be to accelerate diagnosis and provision of services for dementia in resource-poor regions, empowering locals through training programs that include dementia as one component. The partnerships should develop connections with the governments of LMICs and should educate the general population about dementia as a public health problem.

## Conclusions

As infant and childhood mortality rates decline and life expectancies lengthen in LMICs, increases in both total population size and the proportion of elderly individuals will make dementia an increasingly severe burden on healthcare resources. Prevalence of dementia is already high in some developing countries. In Africa, India, and Bangladesh, where prevalence of dementia is currently low, rapidly increasing populations of elderly and increasing prevalence of risk factors, such as HIV-related dementia and traumatic brain injury, foretell a rise in dementia patients that is a disaster waiting to happen. Local governments are unlikely to switch their attention from ongoing problems of malnutrition and communicable disorders to an “incurable” disease like dementia. Furthermore, prevalence of many preventable chronic diseases, such as diabetes and cardiovascular disease, is increasing as LMICs adopt westernized lifestyles, further increasing pressure on limited healthcare resources.

There are currently no preventive measures or cures for dementia. Even if promising immunological approaches, gene therapy, or other strategies turn out to be safe and effective, they are likely to be expensive. Therefore, better management is the most appropriate approach for dealing with dementia in the developing world. However, it appears unlikely that funding agencies will develop initiatives to improve diagnosis of dementia in developing countries in the near future. Thus, it is vital for the international healthcare community to take action now to strengthen existing initiatives, develop North/South and South/South partnerships, and build capacity for dementia diagnosis in resource-poor populations.
